# Role of NFAT in the Progression of Diabetic Atherosclerosis

**DOI:** 10.3389/fcvm.2021.635172

**Published:** 2021-03-11

**Authors:** Yaoyao Cai, Haipeng Yao, Zhen Sun, Ying Wang, Yunyun Zhao, Zhongqun Wang, Lihua Li

**Affiliations:** ^1^Department of Pathology, Affiliated Hospital of Jiangsu University, Zhenjiang, China; ^2^Department of Cardiology, Affiliated Hospital of Jiangsu University, Zhenjiang, China

**Keywords:** NFAT, atherosclerosis, vascular calcification, diabetes, targeted therapy

## Abstract

Nuclear factor of activated T cells (NFAT) is a transcription factor with a multidirectional regulatory function, that is widely expressed in immune cells, including cells in the cardiovascular system, and non-immune cells. A large number of studies have confirmed that calcineurin/NFAT signal transduction is very important in the development of vascular system and cardiovascular system during embryonic development, and plays some role in the occurrence of vascular diseases such as atherosclerosis, vascular calcification, and hypertension. Recent *in vitro* and *in vivo* studies have shown that NFAT proteins and their activation in the nucleus and binding to DNA-related sites can easily ɨnduce the expression of downstream target genes that participate in the proliferation, migration, angiogenesis, and vascular inflammation of vascular wall related cells in various pathophysiological states. NFAT expression is regulated by various signaling pathways, including CD137-CD137L, and OX40-OX40L pathways. As a functionally diverse transcription factor, NFAT interacts with a large number of signaling molecules to modulate intracellular and extracellular signaling pathways. These NFAT-centered signaling pathways play important regulatory roles in the progression of atherosclerosis, such as in vascular smooth muscle cell phenotypic transition and migration, endothelial cell injury, macrophage-derived foam cell formation, and plaque calcification. NFAT and related signaling pathways provide new therapeutic targets for vascular diseases such as atherosclerosis. Hence, further studies of the mechanism of NFAT in the occurrence and evolution of atherosclerosis remain crucial.

## Introduction

Diabetes and atherosclerosis are global public health concerns with an increasing incidence. Atherosclerosis is the leading cause of morbidity and disability death in patients with type 1 and type 2 diabetes. The risk of atherosclerosis in patients with diabetes is also significantly higher than in non-diabetic patients, seriously affecting the lives of patients with diabetes. It also places a huge social, financial and health burden on communities worldwide. According to statistics, there are 488 million people with diabetes in 2019. However, as the leading cause of disability and even death in diabetics, the mechanism of atherosclerosis is very complicated. A large number of molecules and signaling pathways mediate the occurrence and development of atherosclerosis. Nuclear factor-activated T cell 1(NFATc1) is one of five members of the NFAT family, and is widely expressed in cells including various tissues and organs of the vascular wall. In recent years, a growing number of studies have reported key roles for NFATc1 and related signaling pathways in the development of diabetes and initiation of atherosclerosis, such as the phenotypic transformation and migration of vascular smooth muscle cells, endothelial cell injury, macrophage-derived foam cell formation, and plaque calcification. In addition, NFATc3 in NFAT family members also play a role in these processes. NFAT and related signaling pathways represent new therapeutic targets for vascular diseases such as atherosclerosis, providing new hope for the treatment of atherosclerosis and improving the quality of life and survival rate of patients with diabetes. Therefore, the function of NFAT as an important regulator of atherosclerosis and the interventions targeting NFAT and related pathways to prevent atherosclerosis are reviewed and their prospects are discussed.

## Overview of Nuclear Factor of Activated T Cells

### NFAT Family

**Figure 1:**
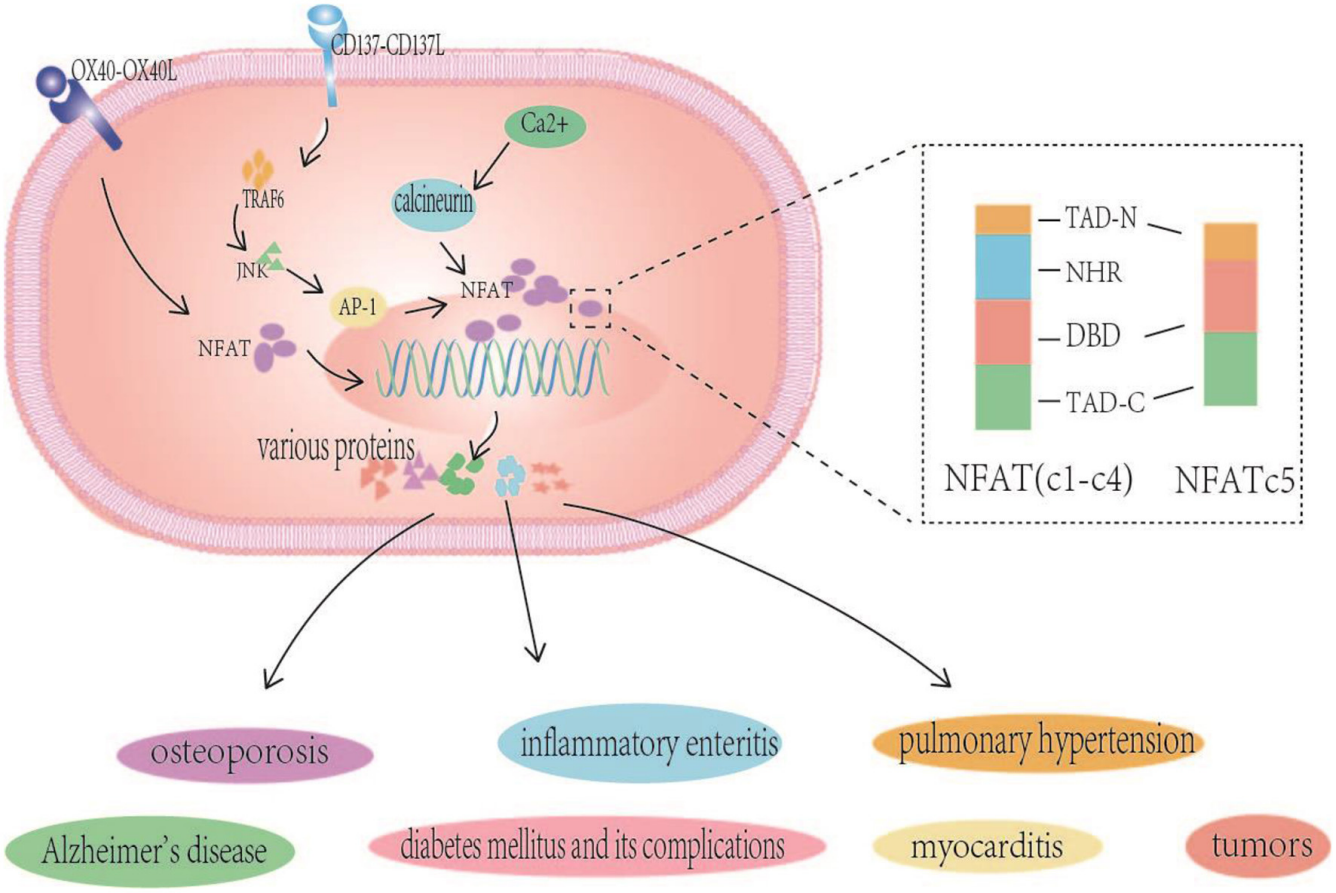
NFAT structure and NFAT related signaling pathways in various human diseases. NFAT (NFATc1-c4) proteins contain two adjacent and highly conserved regions: the NFAT homologous region (NFAT homology region, NHR) and DNA-binding domain (DBD), and the TADs are highly variable regions. NFATc5 is lack of NHR. The inactive highly phosphorylated NFAT protein that is originally located in the cytoplasm interacts with the already activated calcineurin to undergo dephosphorylation and nuclear translocation, inducing NFAT-mediated gene expression, are closely related to associated with many diseases such as Alzheimer's disease, pulmonary hypertension, osteoporosis, inflammatory enteritis, myocarditis, tumors, and diabetes mellitus and its complications. CD137-CD137L signals also alter the expression of NFATc1 through TRAF6/JNK/AP-1 pathways, and OX40-OX40L interaction regulated the expression of lymphocyte NFATc1.

NFAT is a transcription factor with multiple regulatory functions that was initially identified by Shaw et al. in nuclear extracts of activated T cells; NFAT binds to the interleukin 2 promoter, initiating the transcription of genes involved in the immune response and promoting the activation of T cells (1). The molecule is expressed in a variety of other immune and non-immune cells, such as B cells, NK cells, mast cells, mononuclear macrophages and eosinophils, chondrocytes, adipocytes, cardiomyocytes, etc. The NFAT family contains five members: NFAT1 (NFATc2), NFAT2 (NFATc1), NFAT3 (NFATc3), NFAT4 (NFATc4), NFAT5 (NFATc5) (2, 3). The relationship between NFATc1 and the development of diabetic atherosclerosis is closer than to other members of the family. Therefore, the focus of our study is NFATc1, namely, NFAT2 mentioned above. Furthermore, some studies of NFATc3 are described in this section (Figure 1).

### NFAT Structure

NFAT proteins contain two adjacent and highly conserved regions: the NFAT homologous region (NFAT homology region, NHR) and DNA-binding domain (DBD). The DBD is highly conserved with the Rel family protein nuclear factor κB (NF-κB). The Rel homologous regions of NF-κB have similar amino acid sequences, NHR at NFAT amino acid ends, calcineurin, and calcineurin (CaN) binding sites, CaN activates NHR dephosphorylation, exposing the nuclear localization sequence (nuclear localization sequence, NLS) of NFAT and causing its nuclear translocation and activation. The lack of NHR, in NFAT5 is noteworthy, as it is the only family member that is not regulated by the CaN signal. However, calmodulin and NFAT5 have been shown to interact to some extent, and the specific mechanism by which they affect the activity of NFAT5 is currently unclear. NHRs have multiple serine/threonine motifs and are phosphorylated by multiple kinases. NHR rephosphorylation exposes nuclear export signals to the NES to conceal the NLS, and NFAT migrates from the nucleus to the cytoplasm. This reversible phosphorylation controls NFAT shuttling between the nucleus and the cytoplasm and changes its transcriptional activity. Although NFAT members share conserved domains, the TADs are highly variable regions (4, 5).

### NFAT-Related Signaling Pathways and Diseases

Of the many NFAT regulated signaling pathways, the calcineurin- NFAT signaling pathway is the most well-studied. The inactive highly phosphorylated NFAT protein that is originally located in the cytoplasm interacts with the already activated calcineurin to undergo dephosphorylation and nuclear translocation, thereby affecting the expression of subsequent related genes, i.e., inducing NFAT-mediated gene expression, which triggers a series of biological effects of NFAT, such as bone formation, chondrocyte proliferation, cartilage catabolism, vascular formation, and neuropeptide response, T cells activation, expression of cellular inflammatory factors, and angiogenesis. As a result, NFAT levels are closely associated with many diseases such as Alzheimer's disease, pulmonary hypertension, osteoporosis, inflammatory enteritis, myocarditis, tumors, and diabetes mellitus and its complications (5–9). In summary, the association between NFAT and diseases is mainly attributed to its roles in the activation of T cells, immune cell function, the expression of specific genes and promoting the inflammatory response. Emphasis is placed on the relationship between NFAT and diabetes and the initiation of atherosclerosis.

NFAT has been shown to play an important role in the pathogenesis of diabetes and the development of vascular complications. In β cells that secrete insulin, an increase in the cytosolic Ca^2+^ concentration directly stimulates the exocytosis of insulin-containing vesicles. However, calcium also activates multiple signaling pathways, including NFAT, which has important implications for many key cellular processes in β cells. For example, Ca^2+^ mediates insulin transcription through NFAT -dependent pathways and promote the long-term activation of these pathways in cells to impair their normal function and may lead to the onset of type 2 diabetes. Using confocal imaging of immunofluorescence staining, NFAT was shown to be expressed in retinal microvascular endothelial cells, and was readily activated by high sugar. Moreover, *in vivo* inhibition of NFAT decreased the retinal vascular expression of OPN and ICAM-1, prevented diabetes-induced retinal downregulation of the anti-inflammatory cytokine IL-10, and eliminated the phenomenon of increased vascular permeability in diabetic mice. In a diabetic nephrotic model (db/db mice), the ATF3-NFAT axis induced podocyte damage, and ATF3 (activating transcription factor 3) directly regulated NFATc1 gene promoter activity to modify the expression of Wnt6 and Fzd9, which are direct target genes of NFATc1 signals; NFAT also induced podocyte damage through these receptors (10–12). The characteristics of diabetic plaques and the role of NFAT in the mechanism of plaque progression are described below.

## Characteristics of Diabetes Complicated With Atherosclerosis

### Epidemiological Characteristics

Diabetes, a group of metabolic diseases characterized by hyperglycemia, has become a serious global health burden and will become an increasingly serious challenge (13). Trends in the prevalence of increased fasting blood glucose levels and diabetes have more than doubled in the three decades since its initial report in 1980 to 2008. A systematic analysis of health screening surveys and epidemiological studies of 370 countries and 2.7 million participants shows that more than 40% of patients with diabetes reside in China and India, and, as expected, the most populous countries, have the largest number of patients with diabetes (14, 15). According to the global diabetes map from the National Diabetes Federation, ~366 million patients with diabetes were identified worldwide in 2011. Based on this information, Whiting et al. predicted that the number will increase to 552 million by 2030. In 2013, ~382 million people worldwide suffered from diabetes, and by 2035, this number will increase to 590 million. In 2015, 415 million patients with diabetes aged 20–79 years were estimated worldwide, and by 2040, this number will increase to 642 million (16). In 2017, ~451 million patients with diabetes (18–99 years old) were identified worldwide. By 2045, these figures are expected to increase to 693 million (17). In 2019, ~463 million people worldwide had diabetes. By 2045, the number of patients with diabetes is expected to reach 700.2 million (18). The results presented above show a further increase in the global trend of the number of patients with diabetes. Diabetes is getting worse, patients with diabetes are spending increasing amounts on care worldwide, and diabetes has imposed huge social, financial, and health system burdens worldwide. For patients with diabetes, the control of blood glucose levels and, more importantly, the prevention of the complications of diabetes, especially atherosclerosis, which is one of the most dangerous vascular complications of diabetes, are the main goals. Atherosclerosis is characterized by the formation of congee tumors or fibrous plaques in the vascular intima and results in stiffening of the wall, narrowing of the lumen and weakening elasticity; it is the most common disease of the cardiovascular system that causes ischemic changes in the corresponding organ. Atherosclerosis is the main cause of coronary heart disease, cerebral infarction and peripheral artery disease. Fowke et al. systematically reviewed the literature on the prevalence of peripheral artery disease from 1997 to 2010, Based on the results, 202 million people worldwide had peripheral artery disease in 2010, and diabetes is one of the most important risk factors after smoking, Epidemiological evidence shows that diabetes and other risk factors continue to be significantly related to peripheral artery disease (19). Song et al. showed that the global prevalence of peripheral artery disease among people aged 25 and over is 5.56% (95% CI 3.79–8.55) in 2015, the equivalent of 236.62 million people worldwide. Additionally, a meta-analysis confirmed that diabetes and peripheral artery disease are positively correlated (20). Song et al. conducted a meta-analysis of the main risk factors for CAS in Chinese adults, clarifying that diabetes is an important risk factor for atherosclerosis (21). Gedebjerg et al. found in their nationwide DD2 study cohort study that one-third of newly diagnosed patients with T2D were also diagnosed with microvascular and macrovascular complications in the hospital before and after the diabetes diagnosis (22). Ibebuogu et al. reported a higher prevalence of single and multiple vascular diseases in patients with diabetes than in non-diabetic patients (23). Song et al. showed that ~28% of the general population aged 30–79 had abnormal carotid intima-media thickness in 2020, representing more than 1 billion people. In addition, ~21% of people aged 30–79 (816 million) had carotid plaques and 1.5% (58 million) had carotid stenosis. Smoking, diabetes, and hypertension are common risk factors for an increased carotid intima-media thickness and carotid plaques, and early detection and treatment of diabetes and hypertension may help slow the progression of atherosclerotic complications (24). In summary, diabetes and atherosclerosis are diseases that are present in a very large number of people worldwide, and the number of patients increases annually. These diseases may be interconnected or independent. Diabetes is a risk factor for atherosclerosis. Atherosclerosis is also a dangerous and common vascular complication of diabetes. For the foreseeable future, as the diabetes incidence increases, more cases of atherosclerosis, such as peripheral artery disease and carotid plaques, will occur, and more epidemiological research and attention to diabetes and atherosclerosis will improve the quality of life of patients with diabetes.

### Mechanisms of Atherosclerosis in Diabetes Mellitus

Atherosclerosis refers to the deposition of the lipids cholesterol and cholesterol ester in the intima and intima of the artery and its branches, accompanied by the proliferation and migration of smooth muscle cells in the middle layer to the subintima, causing the thickening of the intima and the formation of yellow or grayish yellow plaques resembling porridge tumor-like substances. Atherosclerotic cardiovascular disease is the main cause of death and disability in patients with diabetes, and the risk of atherosclerotic lesions in patients with diabetes is significantly higher than in non-diabetic patients. A large number of clinical and basic studies have confirmed the close relationship between the two diseases. Recent studies have suggested that the mechanism of atherosclerosis in patients with diabetes may involve hyperglycemia, insulin resistance, vascular calcification, oxidative stress, endothelial dysfunction, and the inflammatory response.

#### Characteristics of Diabetic Plaques

The incidence of atherosclerosis in patients with diabetes is significantly higher than in patients without diabetes. Relevant studies have shown that advanced glycation end products are important to factors that promote inflammation, oxidative stress, apoptosis, and the formation of microcalcification foci in the atherosclerotic lesion area of patients with diabetes. Advanced glycation end products promote the transition of plaques from the stable to vulnerable type, eventually leading to rupture and thrombosis that promote the development of acute coronary syndrome and other acute cardiovascular and cerebrovascular events. In addition to advanced glycation end products, fluctuations in blood glucose abnormalities, especially blood glucose levels, are closely associated with atherosclerotic factors such as oxidative stress, inflammation, endothelial dysfunction, and angiogenesis, which also stimulate plaque progression. These mechanisms may be closely related to the morphology and composition of diabetic atherosclerotic plaques. The macrophage plaque area and T cell infiltration in patients with diabetes are significantly higher than in non-diabetic patients (25). Burke et al. observed higher average necrotic core and total plaque and distal plaque loads in patients with type 2 diabetes than in non-diabetic patients, and the size of the necrotic core was positively correlated with the diabetes status. This association may be related to the increase in the number of smooth muscle cells and macrophages expressing RAGE in the plaques of patients with diabetes (26). Jing et al. found a larger plaque lipid core in patients with diabetes, while the fiber cap thickness was relatively thin, which increased plaque instability (27). In addition, diabetes affects related signaling pathways in vascular tissue, leading to vasomotor dysfunction, and the potential acceleration of atherosclerosis (28). The proportion of calcified plaques and mixed plaques in the coronary artery of patients with diabetes was higher than in non-diabetic patients. The increase in the proportion of mixed plaques may partially explain the increase in the coronary heart disease -related mortality rate in patients with diabetes (29). Compared with non-diabetic patients, symptomatic Chinese patients with diabetes are more likely to develop carotid plaque calcification and lipid necrotic cores, more lipid-rich plaques, and calcification. Thus, patients with diabetes may develop more severe atherosclerotic diseases (30). Atherosclerotic plaques occur at sites of intimal calcification, but the more common type of calcification in patients with diabetes is medial calcification. This type of calcification is closely related to the occurrence of cardiovascular adverse events in patients with diabetes (31). Taken together, compared with non-diabetic patients, the vasculature of patients with diabetes is rich in smooth muscle cells and macrophages expressing high levels of RAGE. Vascular endothelial function is affected by long-term hyperglycemia, and the balance between arterial contraction and relaxation is lost. Plaques exhibit more inflammatory cell infiltration, increased inflammatory cell activity and larger lipid necrotic centers, which increases the vulnerability and instability of atherosclerotic plaques.

#### Mechanism of Plaque Formation and Progression

Atherosclerosis is a complex process involving the transformation of multiple cell types and important intercellular interactions. Finally, a series of stages from the initial lipid striation stage to the formation of complex atherosclerotic plaques and secondary lesions increase the chance of cardiovascular adverse events. The exact origin of plaque formation is unclear; however, a large number of clinical and basic studies have shown that endothelial dysfunction is one of the important early contributing factor. Endothelial cells, which form a semipermeable barrier between blood and vascular smooth muscle cells, are essential to maintain vascular homeostasis. By releasing vasoconstrictor and vasodilator substances, the endothelial cell structural integrity ensures permeability and adhesion. Acute hyperglycemia in patients with diabetes may reduce vascular endothelial cell function, reduce the bioavailability of NO, a major vasodilator substances induce the release of inflammatory factors and growth factors, and increase monocyte and leukocyte adhesion to partially mediate oxidative stress and the inflammatory response. The inflammatory mechanism accompanies the whole process of AS development. Although endothelial injury initiates the inflammatory response, chronic inflammation may also lead to or aggravate endothelial cell damage. Upon the stimulation of vascular inflammation, a large number of monocytes attach to the damaged endothelium through a process mediated by inflammatory factor chemotaxis and activation, and then infiltrate into the intima. Finally, with the migration and transformation of middle membrane smooth muscle cells in response to various factors, these cells participate in the formation of complex plaques. Immature neovascularization in plaques may also aggravate lipid deposition and inflammatory cell aggregation, lead to plaque bleeding, make plaques unstable, and increase the incidence of adverse cardiovascular events.

Therefore, with the increase in the number of patients with diabetes and atherosclerotic cardiovascular disease worldwide, studies of the mechanism of atherosclerosis have received increasing attention, including the exploration of genes involved in the molecular mechanism. The NFAT molecule is involved in many pathological processes of atherosclerosis, including foam cell formation, vascular inflammation, VSMC migration, proliferation, phenotypic transformation, vascular calcification, and plaque formation. Shiny et al. reported that the atherogenic effect of hyperinsulinemia on vascular smooth muscle cell migration and proliferation is mediated by mitochondrial dysfunction and the aggregation of oxidative stress signals. Later, increased mRNA expression was observed in monocytes isolated from patients with type 2 diabetes, which was positively correlated with insulin resistance and blood glucose load, indicating that studies of the role of NFAT in the formation of diabetic atherosclerotic plaques have important clinical significance.

## Role of NFAT in Diabetic Plaque Formation and Evolution

AS is a chronic inflammatory vascular disease, as mentioned above, and its pathological process involves many aspects, including endothelial cell dysfunction, the proliferation, and migration of vascular smooth muscle cells, foam cell formation, vascular inflammation. A large number of studies have shown that NFAT molecules play an important role in promoting the formation and evolution of atherosclerosis. The NFATc1 level is significantly higher in patients with coronary heart disease patients than in the control group, and the NFATc1 level in the unstable plaque group is higher than in the stable plaque group. Thus, human NFATc1 is an active index of unstable plaques that can be used to evaluate the risk of coronary heart disease and further predict the occurrence of acute coronary events (32). Therefore, molecules in NFAT-related pathways may also provide the basis for targeted therapy of atherosclerosis and predict the risk of acute cardiovascular events to improve the survival rate and quality of life of patients with diabetes. NFAT is a transcription factor with multiple regulatory functions that is involved in many pathological processes of AS development and is discussed from several perspectives, as listed below.

### Expression of NFAT

**Figure 2:**
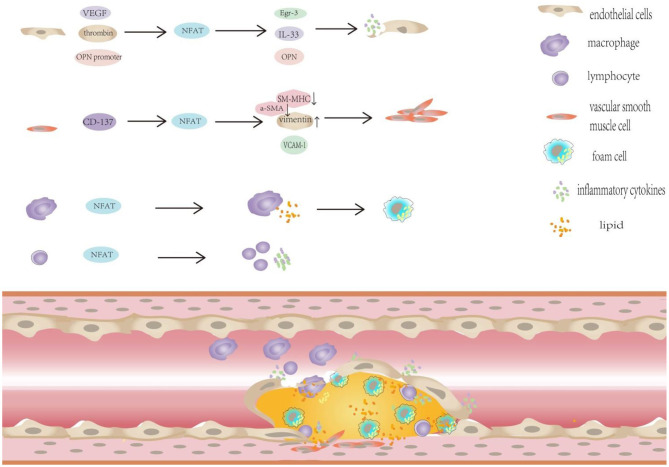
NFAT plays important regulatory role in the progression of diabetic atherosclerosis. NFAT plays important regulatory role in the progression of diabetic atherosclerosis: (1) NFAT and diabetic endothelial dysfunction. ① NFATc1 and NFATc2 participate in mediating VEGF-induced Egr-3 expression in endothelial cells. ② NFATc1 is necessary for thrombin -mediated IL-33 expression. ③ The potential NFAT-dependent regulation of OPN plays an important role in vascular endothelial dysfunction. (2) NFAT promotes VSMC proliferation, migration and phenotypic transformation in patients with diabetes. The activation of CD137 signaling decreased the expression of the contractile markers SM-MHC and α-SMA, but increased the expression of the synthetic phenotypic marker vimentin *in vivo* and *in vitro*. (3) NFAT transcription factors promote macrophage infiltration, foam cell formation.

Recently, an increasing number of studies have investigated the effect of NFAT on atherosclerosis, and the expression of NFAT-related factors is very important for its corresponding role. The study found that CD137-CD137L interaction can regulate the expression of activated T cells and factors in apolipoprotein E knockout mice (33). CD137 molecules regulate NFATc1 expression through miRNA-145a-5p (34). CD137 signaling affects NFATc1 expression in mouse VSMCs through NF-kB p65 (35). The CD137—–CD137L receptor ligand signaling axis is negatively regulated by micro RNA-124-2, thereby affecting NFATc1 expression, Based on these results, mi R-124-2 forms complementary base pairing with the NFATc1 m RNA to induce its degradation. On the other hand, the stability of m RNA translation is affected by its interaction with the NFATc1 3′ untranslated region (36). CD137-CD137L signals also alter the expression of NFATc1 through TRAF6/JNK/AP-1 pathways, and the expression of CD137-CD137L signaling molecules regulates NFATc1 by triggering the TRAF6/JNK/AP-1 pathway (37). CD137 affects NFATc1 expression in vascular smooth muscle through the above signals, then affect gene expression through NFATc1, participates in the phenotypic regulation of vascular smooth muscle cells, and promotes angiogenesis in atherosclerotic plaques (38). A previous study of an Apo E^−/−^ mouse model of atherosclerotic plaques identified a positive correlation between NFATc1 and OX40 and OX40L expression in cervical and splenic lymphocytes; the OX40-OX40L interaction regulated the expression of lymphocyte NFATc1 in Apo E^−/−^ mice (39). Subsequent studies have confirmed that OX40-OX40L signaling regulates NFATc1 expression and affects atherosclerotic plaque formation in mice. However, the specific mechanism needs to be studied (40). Based on these findings, the interaction of CD137L and CD137 plays an important role in the development of atherosclerotic plaques, The OX40-OX40L signaling cascade, which promotes the development of plaques and increases the instability of atherosclerotic plaques, is related to the expression of NFAT in vascular smooth muscle cells and lymphocytes. Hyperglycemia stimulation activates vascular smooth muscle cells NFAT activation (41). Therefore, the study of NFAT especially the specific mechanism by which NFATc1 induces atherosclerosis development, is very important for a better elaboration the mechanism of by which CD137 and OX40-related signals promote AS progression and the mechanism of atherosclerosis caused by diabetes (Figure 2).

### NFAT Transcription Factors and Diabetic Endothelial Dysfunction

The earliest pathological manifestation of atherosclerosis is endothelial cell dysfunction, which is characterized by the production or increased bioavailability of nitric oxide (NO), leading to greater vasoconstriction, inflammation, and thrombosis and an increased susceptibility to atherosclerosis and other microvascular lesions. Therefore, endothelial cell activation and dysfunction are the basis of many vascular diseases and play an important role in AS development. In recent years, impaired endothelial function has been increasingly recognized as an early and harmful marker of various vascular complications of diabetes, including macrovascular complications such as atherosclerosis and microvascular complications such as diabetic nephropathy and retinopathy (42, 43). Many scholars propose that many mechanisms are potentially involved in the pathophysiological process of vascular endothelial cell dysfunction in patients with a long history of diabetes. For example, Meza et al. found that hyperglycemia and T2D are strong stimulators of NOX1- and NOX2 -dependent endothelial dysfunction, but an increasing number of mechanisms and signaling pathways remain to be studied (44). Diabetic hyperglycemia is an effective stimulant that activates calcium (Ca^2+^)/calcineurin-sensitive transcription factors to subsequently activate nuclear factors of activated T cells (NFAT) in arterioles and moderate resistance arteries. NFAT blockade eliminates the exacerbation of atherosclerosis caused by diabetes (45). Therefore, NFAT and vascular endothelial dysfunction may represent targets and a basis for the study of other diabetes related vascular diseases.

#### NFAT Involvement in VEGF-Mediated Induction of Endothelial Egr-3 Expression

Suehiro et al. found that the early human growth-reactive protein (early growth response protein 3, Egr-3) is a key determinant of endothelial cell activation and suggested that Egr-3 may be a new target for vascular disease (46). While vascular endothelial growth factor (VEGF) induces Egr-3 expression in human primary endothelial cells through a process mediated by transcription factors such as NFAT, relevant experiments show that VEGF causes the nuclear localization of NFATc1, NFATc2, and other family members within 15 min. Moreover, VEGF stimulation of Egr-3 expression is significantly reduced by cyclosporine A, a calcineurin/NFAT inhibitor. These results strongly suggest a role for NFATc1 and NFATc2 in mediating VEGF-induced Egr-3 expression in endothelial cells. Overexpression of NFATc1 and NFATc2 causes Egr-3 upregulation and the excessive activation of endothelial cells, which might lead to vascular disease, such as pathological angiogenesis, inflammation, and atherosclerosis. NFAT plays a role in mediating the positive effects of VEGF on EGR-3 expression in endothelial cells. Specifically, two NFAT/NF-B elements in the Egr-3 upstream promoter sequence are necessary for their transcriptional activation, NFATc1 and NFATc2 bind to the region, namely, NFATc mediates the catalytic activation of NFAT/NF-κB elements. Therefore, NFAT is involved in VEGF induced Egr-3 expression in endothelial cells and regulates cell growth, migration, neovascularization, the hemostatic balance, and leukocyte adhesion. These findings provide new insights into the molecular mechanism of endothelial cell activation and dysfunction, and the results suggest that NFAT and Egr-3 may be therapeutic targets in pathological endothelial cell activation- and dysfunction- related diseases.

#### IL-33 Expression Mediated by NFAT and Thrombin

An increase in IL-33 expression was observed in atherosclerotic plaques, and thrombin-induced interleukin-33 expression plays a role in endothelial dysfunction, stimulates vascular endothelial damage, is involved in regulating HASMC proliferation and migration, and promotes vascular wall remodeling. Govatati et al. (47) found that thrombin-induced IL-33 expression requires LMCD1 -enhanced combined activation of NFATc1 and E2F1; from another perspective, NFAT factors are associated with endothelial cell dysfunction. Since the IL-33 promoter region from nt −183 to nt 98 contains only one potential NFAT binding site at nt 100, the authors generated a site mutation, and tested its reactivity to thrombin. The destruction of the NFAT binding site at −100 nt significantly attenuated thrombin-induced IL-33 promoter activity, and NFATc1 was also detected in thrombin-induced protein- DNA complexes. These results suggest that NFATc1 is necessary for thrombin -mediated IL-33 expression. IL-33 has also been identified as another target of NFATc1 that mediates endothelial dysfunction and vascular wall remodeling, providing a new direction for the study of NFAT-related targeted therapy for atherosclerosis.

#### Ca2/NFAT Is Involved in Serum-Induced Endothelial Dysfunction and the Inflammatory Response in Patients With KD

Kawasaki disease (KD) also known as cutaneous and mucosal lymph node syndrome, causes endothelial dysfunction and inflammation, and is the most common systemic vasculitis syndrome in children. The expression levels of NFATc1, NFATc3, and inflammatory molecules (e.g., E- selectins, VCAM-1, TF, and MCP-1) were increased in their endothelial cells treated with patient serum. In addition, a genetic analysis identified, 16 mutations in the key genes of the Ca2/NFAT signaling pathway in children with KD, and these mutations are closely associated with KD-related vasculitis. These results further suggest that Ca2/NFAT is involved in serum-induced endothelial dysfunction and the inflammatory response in patients with KD, and thus participates in the pathogenesis of KD-related vasculitis. The inflammatory response induced by cyclosporine (CsA) mediated Ca^2+^/NFAT inhibition reduces endothelial dysfunction. CsA inhibits inflammation and reduces the dysfunction of coronary endothelial cells by regulating Ca^2+^/NFAT, it exerts an important cytoprotective effect. Wang et al. postulated that NFAT may represent a new marker for the clinical prediction of KD, and NFAT inhibitors are also expected to provide new insights into and drugs for the clinical treatment of KD. Many KD drugs targeting the Ca^2+^/NFAT signaling pathways have been reported, such as cyclosporine A (CsA) and FK506, which are NFAT targeting drugs (48). Thus, we suggest that the vascular complications observed in patients with diabetic may be similar to the mechanisms described above. Certain serum components (possibly VEGF or other inflammatory molecules) in patients with diabetes induce Ca^2+^/NFAT activation in endothelial cells and endothelial cell dysfunction, which may be one of the causes of vascular complications. Considering this hypothesis, we can apply drugs targeting the Ca^2+^/NFAT signaling pathway to the treatment and maintenance of diabetic atherosclerotic complications accompanied by arterial endothelial cell dysfunction or disorder.

#### NFAT-Dependent Regulation of OPN and Vascular Endothelial Dysfunction

As shown in the study by Zetterqvist et al., the level of osteopontin (OPN) in the aorta of diabetic mice was increased compared with the control group. After inhibiting NFAT for 4 weeks, the expression levels of OPN and ICAM-1 in the retinal vascular basement were decreased, and inhibition of NFAT *in vivo* eliminated the increase in vascular permeability induced by diabetes. During the experiment, NFAT inhibition decreased OPN mRNA levels in retinal vessels, regardless of diabetes. More directly, hyperglycemia induced the production of the proinflammatory cytokine OPN in the mouse aorta by promoting the direct binding of nfatc3 to the OPN promoter (11). Therefore, it is a reasonable hypothesis is that the potential NFAT-dependent regulation of OPN plays an important role in vascular endothelial dysfunction, leading to increased vascular permeability in patients with diabetes.

In fact, *in vivo* studies have shown that hyperglycemia-induced NFAT activation is associated with microcirculatory endothelial dysfunction, and that therapeutics blocking NFAT significantly improves microcirculatory endothelial function in subjects with diabetes (49). Therefore, further exploration of NFAT inhibitors in the treatment of diabetic vascular dysfunction has certain potential.

### NFAT Promotes VSMC Proliferation, Migration, and Phenotypic Transformation in Patients With Diabetes

The proliferation and migration of VSMCs are an important processes involved in AS development. When blood vessels are damaged, endothelial cells release growth factors and inflammatory factors to stimulate the transition of middle membrane VSMCs from a static state to a proliferative state. The proliferative state is characterized by accelerated migration, proliferation, and production of extracellular matrix components. When VSMCs migrate from the middle membrane to the intima, they increase the thickness of the intima and aggravate the vascular intimal hyperplasia reaction in the AS lesion. Many studies have shown a VSMC phenotypic transformation in individuals with diabetes, and DM2 induces the phenotypic transformation of vascular smooth muscle cells, which is related to hyperglycemia, hyperinsulinemia, and higher levels of advanced glycation end products *in vivo* (50). At the cell level, the activation of platelets and their interaction after endothelial cellular injury are involved in the phenotypic transformation of vascular smooth muscle cells in individuals with diabetes (51). Therefore, studies exploring the effect of NFAT transcription factor activation on VSMCs to elucidate the role of NFAT in the development of diabetic atherosclerosis are very important. NFATc1 is the subtype with the highest expression in smooth muscle cells, along with a small amount of NFATc3, but western blotting does not detect NFATc2; thus studies mainly analyze-NFATc1.

Mancarella et al. found that Ca^2+^-sensing stromal interaction molecule (STIM)-regulated Ca^2+^ homeostasis is essential for NFAT-mediated transcriptional control of SMC proliferation, development and growth in response to injury. STIM1 is required for store-dependent Ca^2+^ influx and the refilling of intracellular Ca^2+^ stores in proliferating SMCs. The continuous increase in Ca^2+^ concentrations activated the nuclear translocation of NFAT through a process mediated by calcineurin, thus inducing the proliferation of smooth muscle cells (52).

Experimental data reported by Zhong et al. show that CD137 regulates the phenotype of vascular smooth muscle cells and that NFATc1 knockout inhibits the increase in CD137 induced migration of vascular smooth muscle cells, suggesting that NFATc1 plays an important role in the phenotypic regulation of vascular smooth muscle cells induced by the CD137-CD137L interaction (53). The activation of CD137 signaling decreased the expression of the contractile markers SM-MHC and α-SMA, but increased the expression of the synthetic phenotypic marker vimentin *in vivo* and *in vitro*. Therefore, the role of CD137 in atherosclerosis may be related to vascular smooth muscle cell phenotype. Moreover, immunofluorescence staining showed that CD137 not only upregulated the expression of the NFATc1 protein but also promoted nuclear translocation of NFATc1. NFATc1 overexpression in vascular smooth muscle cells significantly affects the cell phenotype. Orr and other studies have found that calcineurin/NFAT signaling regulates VCAM-1 expression induced by atherosclerotic monomers. Vascular cell adhesion molecule-1 (VCAM-1) expression is necessary for smooth muscle cell migration and proliferation, and is also involved in the phenotypic transformation of smooth muscle cells, playing an important role in the progression of atherosclerosis (54). According to Cheng et al., Klf-5 is an important regulator of phenotypic transformation, but they only confirmed the effect of this factor on α-SMA expression. In their study, NFATc1 affected α-SMA expression and significantly altered the expression of SM-MHC and vimentin, the levels of these phenotypic markers may be an independent key regulator of the phenotypic regulation (55). NFATc1 increased and decreased the expression of VSMC differentiation marker genes through direct or indirect effects, which increased the migration of these cells, eventually forming neointimal lesions. Activated CD137 signaling regulates the expression of NFATc1 and its downstream factors in vascular smooth muscle cells through TRAF6/NF-kB p65 pathways, providing a new target for atherosclerosis therapy (56).

In addition, Pang et al. also found that the activation of calcineurin and the nuclear translocation of NFATc1 are necessary for PE to induce the proliferation of vascular smooth muscle cells. As shown in our previous study, CsA inhibits calcineurin activity and NFATc1 nuclear translocation, partially inhibits PE-induced vascular smooth muscle cell proliferation, and more clearly inhibits the calcineurin-NFATc1 pathway involved in phenylephrine induced vascular smooth muscle cell proliferation (57). More specifically, Shiny et al. observed that 1 hyperinsulinemia increased NFATc1 expression and nuclear translocation in vascular smooth muscle cells and insulin therapy resulted in the increased proliferation and migration of vascular smooth muscle cells compared with untreated controls. NFATc1 overexpression increases VSMC migration and proliferation due to hyperinsulinemia. Treatment with 11 R-VIVIT or cyclosporine A significantly decreased insulin-mediated migration of vascular smooth muscle cells, increased transcription in vascular smooth muscle cells mediated by NFAT, NOD, and hyperinsulinemia, and NFAT inhibitors reduce hyperinsulinemia and NOD ligand-induced inflammatory responses in vascular smooth muscle cells (58). Therefore, hyperinsulinemia in patients with diabetes increases the expression and activation of NFAT in vascular smooth muscle cells, and NFAT plays a key role in mediating the hyperinsulinemia induced proliferation and migration of vascular smooth muscle cells. Selective NFAT inhibitors may be an effective strategy to coordinate the inhibition of insulin-mediated proliferation and inflammatory responses as well as innate immune changes in vascular smooth muscle cells induced by atherosclerosis.

### NFAT Transcription Factors Promote Macrophage Infiltration, Foam Cell Formation, and Osteoclasto-Genesis

Blood lipid disorder and oxidative inflammation promote monocyte accumulation and their transformation into macrophages to phagocytose lipids under the damaged vascular intima, resulting in the accumulation of a large number of lipid droplets in the cell to form foam cells, which is the central cause of plaque formation. At the same time, these cells secretes a large number of inflammatory mediators, causes inflammatory reaction, affects the stability of arterial plaques, and subsequently induce cardiovascular and cerebrovascular events. High levels of reactive oxygen species (ROS) are detected in patients with diabetes. Excessive ROS activates proinflammatory transcription factors such as NF-κB and AP-1, that upregulate the expression of pro-inflammatory chemokines/cytokines and adhesion molecules, activate endothelial cells, and attract monocytes. These monocytes further exacerbate inflammation, thereby promoting macrovascular and microvascular injury and atherosclerosis along with various vascular complications of diabetes. Diabetes also triggers the formation of ROS in macrophages. According to recent studies, hyperglycemia leads to mitochondrial dysfunction in endothelial cells and monocytes and macrophages, as well as abnormal activation of cytosolic NADPH oxidase (NOX). Macrophages promote inflammation by releasing proinflammatory cytokines and proteases and aggravating vascular lesions (59). Thus, macrophages also play an important role in the progression of diabetic atherosclerosis, and the activation of NFAT transcription factors may be an indispensable factor contributing to this process.

The inhibition of NFAT expression may be an important step in the regulation of certain bioactive substances to ameliorate the progression of atherosclerosis. Green tea protects against atherosclerosis (60). A major active ingredient in green tea is epigallocatechin-3-gallate (EGCG). Krishnan et al. suggest the antiatherosclerotic effect of EGCG may be effect may be mediated by the inhibition of inflammation associated with hepatic steatosis, especially the fusion of macrophages. Compared with NF-κB, EGCG significantly reduced the expression of NF-AT mediated by hepatic TNF-α in hypercholesterolemic rats and the expression of cell adhesion factors such as ICAM-1 and E- selectin, significantly inhibited macrophage infiltration (61). Thus, EGCG may exert a protective effect on atherosclerosis by reducing hypercholesterolemia-induced stress and the infiltration of macrophages induced by ICAM and E- selectin through the NFAT dependent pathway, Mononuclear macrophages in other parts of the body in non-hepatic areas may also be affected, At some extent, EGCG can be used as the basis for targeted inhibition of NFAT expression and antiatherosclerotic drug therapy.

In addition, the mechanism by which iron hemoglobin FHb inhibits the osteoclast differentiation of macrophages in atherosclerotic plaques is also closely related to NFAT, Zavaczki et al. found that FHb (rather than iron hemoglobin) reduced bone resorption activity induced by RANKL (nuclear factor-κ receptor activator) and inhibited osteoclast-specific gene expression (anti-tartaric acid phosphatase, calcitonin receptor and dendritic cell-specific transmembrane protein). Increased osteoblasts and decreased osteoclast activities may contribute to intimal calcification, FHb inhibits NFATc1 nuclear translocation, and thus inhibit RANKL—induced macrophage osteoclast differentiation, FHb plays a certain role in inhibiting the calcification of atherosclerotic plaques (62).

### NFAT Transcription Factor Activation and Vascular Inflammation

The chronic inflammatory theory of the AS mechanism proposes that the inflammatory mechanism is activated throughout initiation, progression, and complications of AS lesions. As a pathological process, chronic inflammation leads to endothelial cell injury, endothelial cell dysfunction, secretion of proinflammatory cytokines, promotion of monocyte adhesion and migration to the endothelium, and phagocytosis of ox-LDL, and other foam cells. Aggravation of local inflammatory reactions accelerates the progression of vascular atherosclerosis. NFAT plays an important role in T cell-mediated transcriptional regulation of genes involved in inflammation and the immune response, and its activation induces the expression of various cytokines, such as IL-2, IL-4, IL-6, IL-8, IL-10, IFN-γ, TNF-α, and CD40L, all of which have been shown to contribute to atherosclerosis. Inflammation is closely related to diabetes. This association exists in two aspects. On the one hand, chronic inflammation appears to promote diabetes. On the other hand, diabetes promotes the inflammatory response and mediates vascular dysfunction, cardiovascular disease, and end-organ damage. Therefore, some researchers postulate that strategies targeting inflammation can improve blood glucose control and reduce vascular complications in patients with diabetes (63). Therefore, studies exploring the mechanism of NFAT and its participation in vascular inflammation and identifying therapeutic targets for the control and prevention of diabetic atherosclerosis and other vascular complications are very important. The results of a study on the upstream signals of IL-6 inflammatory factors in Xinjiang Hazak patients with hypertension in Xinjiang also suggest that voltage-gated potassium channels may cause inflammatory cell release by activating the phosphorylation/activation of NFAT signaling. The release of inflammatory cells is closely related to the mechanism of atherosclerosis, NFAT -related signals and vascular inflammation-related signals (64). OX40-OX40L and NFATc1 may play an important roles in lymphocyte proliferation and atherosclerotic plaques. Studies by Yan et al. have found a significant increase in the plaque surface area and lymphocyte proliferation upon the interaction of OX40-OX40L (65), and blocking OX40-OX40L interactions or the administration of NTATc1 inhibitors (CsA) similarly inhibits cell proliferation and decreases the plaque surface area. Based on these results, OX40-OX40L and NFATc1 may play important roles in lymphocyte proliferation and atherosclerotic plaques. This study also supports the hypothesis that OX40-OX40L interactions can increase the proportion of Th1 cells among lymphocytes and that Th1 cells promote atherosclerosis. NFATc1 is one of the downstream signaling intermediates, and OX40-OX40L may be a good target for future atherosclerosis therapy. In addition, the effect of the oxidative low-density lipoprotein (OXLDL) on NFAT is mediated by oxidative stress, and oxidative stress, which in turn activates the NFAT transcription factors in the calcineurin signaling pathway. This effect of OXLDL and the induction of the inflammatory response also suggest that NFAT may be related to the inflammatory process in atherosclerotic lesions (66). Diabetes-induced hyperglycemia activates NFAT, especially NFATc3, in vascular smooth muscle cells, leading to increased intravascular expression of osteopontin (OPN). OPN is a key regulator of chronic inflammatory diseases, promotes the proliferation of vascular smooth muscle cells, and promotes vascular inflammation and the production of the atherogenic cytokine interleukin-6 (IL-6) (67). In the study by Nilsson-Berglund, STZ—induced diabetes increased OPN expression in the ascending and thoracic aorta, the vascular segments of patients with diabetes were particularly prone to atherosclerosis, which was prevented by using NFAT inhibitors *in vivo* or by depriving mice of NFATc3 proteins. Thus, NFATc3 may be associated with diabetic vascular dysfunction.

In addition, CD14 has been shown to activate NFAT, regulate the life cycle of myeloid cells in a TLR4-independent manner, transport inflammatory lipids and induce excessive activation of phagocytes, which plays a key role in mediating inflammation, atherosclerosis and other related diseases. Based on accumulating evidence, CD14 not only functions as a proinflammatory mediator, but also exerts a positive or negative effect on the occurrence and consequences of inflammation by activating its signaling cascade, forming an inflammatory environment in the intima of blood vessels, and directly promoting the formation of atherosclerotic lesions by regulating the fate of inflammatory cells and smooth muscle cells. NFAT participates in one of the mechanisms of atherosclerotic development, which implies the close relationship between NFAT and vascular inflammation (68).

### NFAT Transcription Factor Activation and Lipid Peroxidation

Oxidized low -density lipoprotein (oxLDL) plays a key role in the occurrence and development of diabetic atherosclerosis. Atherosclerosis may be considered an inflammatory disease, and its pathogenesis is related to abnormal lipid metabolism. The transcription factor NFAT (nuclear factor that activates T cells) plays an important role in controlling the expression of cytokine genes involved in the inflammatory response. Mazière et al. showed that OxLDL activated NFAT, which was mediated by increased intracellular calcium concentrations and blocked by the calcineurin inhibitor cyclosporin A. The effect of oxLDL on NFAT is mediated by oxidative stress, which then activates the calcium- calmodulin phosphate signaling pathway of NFAT. OxLDL increases the intracellular ROS level (66). In addition, CD14 activates NFAT, and CD14 was expressed at significantly higher levels in macrophages present in atherosclerotic lesions than in normal intima. The amplified CD14-dependent signaling pathway accelerates the formation of foam cells by increasing the expression of oxidized low-density lipoprotein receptor SR-AI and promotes the adhesion and migration of inflammatory cells into atherosclerotic lesions (68). Based on these results, NFAT was closely related to lipid peroxidation and other metabolic abnormalities.

### NFAT Transcription Factor Activation Promotes Angiogenesis in Diabetic Atherosclerotic Plaques

Intraplaque angiogenesis plays an extremely important role in the pathophysiological mechanisms of atherosclerosis, and NFATc1 promotes neointima formation and atherosclerosis. Angiogenesis is a process involving endothelial cell proliferation, migration, and vessel formation and is a characteristic component of atherosclerosis. A study assessing the incidence of subclinical carotid atherosclerosis showed that it is very common in patients with diabetes (69). Carotid angiography (CEUS) shows that a considerable part of carotid plaque in diabetic patients contains plaque neovascularization, which may be one of the markers of vulnerable plaque, which will lead to an increased risk of plaque rupture. Similarly, Xiong et al. used contrast-enhanced ultrasound to explore the relationship between carotid plaque neovascularization and diabetes mellitus (DM) and found that the enhancement intensity of carotid plaques in patients with diabetes was greater than in non-diabetic patients. Therefore, carotid plaques in patients with diabetes may display higher levels of neovascularization and may be more vulnerable (70). Therefore, we propose that angiogenesis in plaques detected by carotid angiography may develop into a target for monitoring therapeutic strategies for stable atherosclerotic plaques. Even the control of angiogenesis through related mechanisms may improve plaque stability.

The CD137 upregulated NFATc1, in mice and not only induced vascular smooth muscle cell migration, but also induced angiogenesis. The authors proposed a certain correlation between the two mechanisms. Moreover, Weng et al. observed increased angiogenesis in atherosclerotic plaques from Apo E/mice treated with agonist anti- CD137 antibodies, suggesting that activated CD137 signaling induces angiogenesis, endothelial cell proliferation and endothelial cell migration (71). Furthermore, the blockade of CD137 signaling with inhibitory CD137 antibodies inhibits the activation of this pathway, and attenuates agonist anti- CD137 antibody-induced angiogenesis. CD137 signaling regulates Smad1/5 and NFATc1 expression in endothelial cells, Smad1/5 silencing inhibits NFATc1 expression and angiogenesis induced by CD137 signaling, and inhibition of NFATc1 genes inhibits CD137 signal-induced angiogenesis. Therefore, we identified a Smad1/5 pathway for CD137 that induces Smad1/5 phosphorylation and p-Smad1/5 nuclear translocation, and promotes the expression of NFATc1 and the conclusion of transposition. In this pathway, CD137, Smad1/5, and NFATc1 can be used potentially represent as very promising therapeutic targets for the treatment and control of atherosclerotic plaque stability. Activated CD137 signaling also regulates the expression of NFATc1 and its downstream factors in vascular smooth muscle cells through TRAF6/NF-κB p65 pathways, providing a new target for atherosclerosis treatment.

### NFATc1 and Calcifications of Diabetic Atherosclerotic Plaque

Vascular calcification is an abnormal process of active calcium deposition in the vascular wall mediated by mesenchymal cells that differentiated from smooth muscle cells. The phenotypes of osteoblasts, chondrocytes and osteoclasts have been detected in the calcified vascular wall, and even intact lamellar bone and regenerated bone marrow have been observed. Diabetic vascular calcification is characterized by early arterial mesangial calcification and subsequent atherosclerotic plaque calcification, mainly intimal calcification, in which some mechanism of evolution may exist. The NFAT signaling pathway has consistently been shown to be closely related to osteoclasts and diabetic vascular calcification. More studies on the roles of OXLDL- and NFAT- related signaling pathways in this process have been conducted. Goettsch et al. were the first to show that the NFAT signaling pathway is a new regulator of OXLDL-induced differentiation of vascular smooth muscle cells to osteoblast-like phenotype that promotes vascular calcification. Their experiments showed that OXLDL significantly increased NFATc1 and NFATc2 mRNA expression in mature osteoblasts. Moreover, the inhibition of NFAT activity completely prevented the ox LDL-induced osteogenic differentiation of HCASMCs (coronary vascular smooth muscle cells). The authors proposed that NFAT inhibition may be a therapeutic strategy to prevent cellular transdifferentiation during oxidative stress-induced vascular calcification (72). The ability of Osx (bone regulators) to induce bone formation is enhanced by its interaction with nuclear factors that activate T cell c1 (nfaTc1). NFATc1 is important for Osx transcriptional activity. Osx is a key transcription factor involved in the terminal differentiation of osteoblasts, and both activated forms are translocated to the nucleus and may be jointly involved in the process of calcified aortic stenosis (73). The formation of RANK (NF-κB receptor activator)- TRAF6 (tumor necrosis factor receptor-related factor 6) complexes activates NF-κB and AP-1, By regulating NFATc1, these complexes promote osteoclast formation and affect vascular calcification (74). FHb inhibits RANKL—induced osteoclast differentiation in macrophages, suggesting that FHb accumulation in calcified areas of atherosclerotic lesions may delay OLC (osteoclast-like cell) formation and could impair calcium absorption, thereby promoting vascular calcification (62). Moreover, CD137 signals can regulate vascular calcification through the CD137-NFATc1 axis. Excitatory CD137 signals may mediate the bone-like phenotypic transformation and calcification through exocrine signaling, and NFATc1 is the key factor involved in this process (75).

### NFAT-Targeted Therapy

Given the important role of calcineurin-NFAT signaling in various physiological and immune processes, its inhibition is considered a powerful treatment for graft rejection, autoimmune diseases, and cardiovascular diseases. Therefore, the main idea of NFAT research and targeted therapy is to inhibit the activity of calcineurin. The discovery that the immunosuppressants CsA (cyclosporine A) and FK506 (tacrolimus) disrupt calcineurin activity and form CsA procyclin or FK506-FKBP12 complexes also facilitated follow-up studies on various aspects of NFAT factors11. However, these immunosuppressants inhibit and affect all downstream signaling pathways of calcineurin, and lead to with adverse side effects and toxicity toward non-immune cells, limiting their clinical application. Therefore, the development of NFAT inhibitors with greater selectivity and less toxicity direct target NFAT functions is important. In addition to the direct inhibition of calcineurin, another strategy is to prevent calcineurin from interacting with NFAT proteins, selectively inhibiting NFAT activation, by activated calcineurin through an interaction at several docking sites before NFAT dephosphorylation and activation. Specific disruption of the binding of calcineurin to NFAT will effectively inactivate NFAT, without impairing the activity of calcineurin. The selective active peptide developed by Yu et al. (VIVIT) can significantly and more selectively inhibits calcium/calmodulin-activated NFATc1, NFATc2, and NFATc3 dephosphorylation and NFATc2 nuclear translocation, indicating that it may become a promising drug to study the exact role of the NFAT response in atherosclerosis and subsequent targeted therapy. In addition, the effects NFAT on AS mentioned above involve many signaling pathways; hence, these pathways can also be used as targets to influence NFAT activity (76). Thrombin—induced self—inhibitory factor, critical area of Down syndrome-1 (DSCR-1), can attenuate NFAT dependent vascular cell adhesion molecule-1 expression and endothelial cell inflammation, suggesting that DSCR-1 mediated inhibition of NFAT signaling may exert therapeutic effects on the inflammatory state of atherosclerotic plaques (77). Tacrolimus reduces the proliferation of vascular smooth muscle cells and decreases calcineurin, NFATc4, and IL-2 expression. In addition, the newly developed drug TES inhibits intimal hyperplasia after stent placement through calcineurin/NFAT/IL-2 signaling, which is one of several mechanisms for by which TES inhibits restenosis. Calcineurin and NFAT may be important molecular targets to prevent restenosis after stenting (78). Garcia Vaz et al. found that treatment of diabetic mice with the NFAT blocker a-285222 reduced the nuclear accumulation of nfatc3 and NFAT luciferase transcription activity in skin microvessels, thus improving microvascular function. Treatment with a-285222 increased NOS expression in the dermis and the level of NOS in the plasma of diabetic mice. It also prevented the production of the inflammatory cytokines interleukin-6 and osteopontin, reduced plasma endothelin-1 levels and blood pressure, and improved the survival rate of mice without affecting blood glucose levels (49). Zetterqvist et al. also used the NFAT blocker a-285222 in related experiments, indicating that the inhibition of NFAT may exert a protective effect on the retina of diabetic mice (11). Therefore, the inhibitory effect of a-285222 on atherosclerotic deterioration and its complications strongly indicates that the inhibition of NFAT *in vivo* may represent a new treatment method to preserve endothelial function in patients with diabetes mellitus, but further explorations of the potential of NFAT inhibitors as a new treatment method for diabetic microvascular dysfunction are needed.

## Conclusions and Perspectives

In summary, NFAT and its activation play important roles in the development of AS in patients with diabetes. Based on accumulating evidence, NFAT is closely related to the phenotypic transformation and migration of vascular smooth muscle cells, endothelial cell injury, macrophage-derived foam cell formation, and plaque calcification, but little research has been conducted on various aspects of NFAT-centered and NFAT-targeted therapy for AS drug development. Therefore, future studies should focus on determining the various aspects of mechanism by which the NFAT signaling pathway leads to AS formation, and which drugs can affect the expression NFAT vascular wall, thus providing a new method for the clinical treatment of AS-ralated diseases.

## Author Contributions

All authors contributed to the conception and wrote the review, critically reviewed all parts of the manuscript, accepted its final version prior to submission, and account for its content.

## Conflict of Interest

The authors declare that the research was conducted in the absence of any commercial or financial relationships that could be construed as a potential conflict of interest.

## References

[1] Shaw JP Utz PJ Durand DB Toole JJ Emmel EA Crabtree GR. Identification of a putative regulator of early T cell activation genes. Science. (1988) 241:202–5. 10.1126/science.3260404 3260404

[2] Rao A Luo C Hogan PG. Transcription factors of the NFAT family: regulation and function. Annu Rev Immunol. (1997) 15:707–47. 10.1146/annurev.immunol.15.1.707 9143705

[3] Mognol GP Carneiro FR Robbs BK Faget DV Viola JP. Cell cycle and apoptosis regulation by NFAT transcription factors: new roles for an old player. Cell Death Dis. (2016) 7:e2199. 10.1038/cddis.2016.97 27100893 PMC4855676

[4] Aramburu J Lopez-Rodriguez C. Regulation of inflammatory functions of macrophages and T lymphocytes by NFAT5. Front Immunol. (2019) 10:535. 10.3389/fimmu.2019.00535 30949179 PMC6435587

[5] Shou J Jing J Xie J You L Jing Z Yaoa J. Nuclear factor of activated T cells in cancer development and treatment. Cancer Lett. (2015) 361:174–84. 10.1016/j.canlet.2015.03.005 25766658

[6] Qin JJ Nag S Wang W Zhou J Zhang WD Wang H. NFAT as cancer target: mission possible? Biochim Biophys Acta. (2014) 1846:297–311. 10.1016/j.bbcan.2014.07.009 25072963 PMC4710172

[7] Yang Y Chung MR Zhou S Gong X Xu H Hong Y. STAT3 controls osteoclast differentiation and bone homeostasis by regulating NFATc1 transcription. J Biol Chem. (2019) 294:15395–407. 10.1074/jbc.RA119.010139 31462535 PMC6802509

[8] Rojanathammanee L Puig KL Combs CK. Pomegranate polyphenols and extract inhibit nuclear factor of activated T-cell activity and microglial activation in vitro and in a transgenic mouse model of Alzheimer disease. J Nutr. (2013) 143:597–605. 10.3945/jn.112.169516 23468550 PMC3738232

[9] Chen R Yan J Liu P Wang Z Wang C Zhong W. The role of nuclear factor of activated T cells in pulmonary arterial hypertension. Cell Cycle. (2017) 16:508–14. 10.1080/15384101.2017.1281485 28103134 PMC5384583

[10] Sabatini PV Speckmann T Lynn FC. Friend and foe: β-cell Ca^2+^ signaling and the development of diabetes. Mol Metab. (2019) 21:1–12. 10.1016/j.molmet.2018.12.007 30630689 PMC6407368

[11] Zetterqvist AV Blanco F Öhman J Kotova O Berglund LM de Frutos Garcia S. Nuclear factor of activated T cells is activated in the endothelium of retinal micro vessels in diabetic mice. J Diabetes Res. (2015) 2015:428473. 10.1155/2015/428473 25918731 PMC4396720

[12] Zhang H Liang S Du Y Li R He C Wang W. Inducible ATF3-NFAT axis aggravates podocyte injury. J Mol Med. (2018) 96:53–64. 10.1007/s00109-017-1601-x 29038896 PMC5760612

[13] Danaei G Finucane MM Lu Y Singh GM Cowan MJ Paciorek CJ. National, regional, and global trends in fasting plasma glucose and diabetes prevalence since 1980: systematic analysis of health examination surveys and epidemiological studies with 370 country-years and 2.7million participants. Lancet. (2011) 378:31–40. 10.1016/S0140-6736(11)60679-X 21705069

[14] Whiting DR Guariguata L Weil C Shaw J. IDF diabetes atlas: global estimates of the prevalence of diabetes for 2011 and 2030. Diabetes Res Clin Pract. (2011) 94:311–21. 10.1016/j.diabres.2011.10.029 22079683

[15] Guariguata L Whiting DR Hambleton I Beagley J Linnenkamp U Shaw JE. Global estimates of diabetes prevalence for 2013 and projections for 2035. Diabetes Res Clin Pract. (2014) 103:137–49. 10.1016/j.diabres.2013.11.002 24630390

[16] Ogurtsova K da Rocha Fernandes JD Huang Y Linnenkamp U Guariguata L Cho NH. IDF diabetes Atlas: global estimates for the prevalence of diabetes for 2015 and 2040. Diabetes Res Clin Pract. (2017) 128:40–50. 10.1016/j.diabres.2017.03.024 28437734

[17] Cho NH Shaw JE Karuranga S Huang Y da Rocha Fernandes JD Ohlrogge AW. IDF Diabetes Atlas: Global estimates of diabetes prevalence for 2017 and projections for 2045. Diabetes Res Clin Pract. (2018) 138:271–81. 10.1016/j.diabres.2018.02.023 29496507

[18] Saeedi P Petersohn I Salpea P Malanda B Karuranga S Unwin N. Global and regional diabetes prevalence estimates for 2019 and projections for 2030 and 2045: results from the International Diabetes Federation Diabetes Atlas, 9th edition. Diabetes Res Clin Pract. (2019) 157:107843. 10.1016/j.diabres.2019.107843 31518657

[19] Fowkes FG Rudan D Rudan I Aboyans V Denenberg JO McDermott MM. Comparison of global estimates of prevalence and risk factors for peripheral artery disease in 2000 and 2010: a systematic review and analysis. Lancet. (2013) 382:1329–40. 10.1016/S0140-6736(13)61249-0 23915883

[20] Song P Rudan D Zhu Y Fowkes FJI Rahimi K Fowkes FGR. Global, regional, and national prevalence and risk factors for peripheral artery disease in 2015: an updated systematic review and analysis. Lancet Glob Health. (2019) 7:e1020–30 10.1016/S2214-109X(19)30255-4 31303293

[21] Song P Xia W Zhu Y Wang M Chang X Jin S. Prevalence of carotid atherosclerosis and carotid plaque in Chinese adults: a systematic review and meta-regression analysis. Atherosclerosis. (2018) 276:67–73. 10.1016/j.atherosclerosis.2018.07.020 30036743

[22] Gedebjerg A Almdal TP Berencsi K Rungby J Nielsen JS Witte DR. Prevalence of micro and macrovascular diabetes complications at time of type 2diabetes diagnosis and associated clinical characteristics: a cross-sectional baseline study of 6958 patients in the Danish DD2 cohort. J Diabetes Compl. (2018) 32:34–40. 10.1016/j.jdiacomp.2017.09.010 29107454

[23] Ibebuogu UN Nasir K Gopal A Ahmadi N Mao SS Young E. Comparison of atherosclerotic plaque burden and composition between diabetic and non-diabetic patients by noninvasive CT angiography. Int J Cardiovasc Imaging. (2009) 25:717–23. 10.1007/s10554-009-9483-9 19633998

[24] Song P Fang Z Wang H Cai Y Rahimi K Zhu Y. Global and regional prevalence, burden, and risk factors for carotid atherosclerosis: a systematic review, meta-analysis, and modelling study. Lancet Glob Health. (2020) 8:e721–9. 10.1016/S2214-109X(20)30117-0 32353319

[25] Virmani R Burke AP Kolodgie F. Morphological characteristics of coronary atherosclerosis in diabetes mellitus. Can J Cardiol. (2006) 22(Suppl. B):81–4B. 10.1016/S0828-282X(06)70991-6 16498517 PMC2780829

[26] Burke AP Kolodgie FD Zieske A Fowler DR Weber DK Varghese PJ. Morphologic findings of coronary atherosclerotic plaques in diabetics: a postmortem study. Arterioscler Thromb Vasc Biol. (2004) 24:1266–71. 10.1161/01.ATV.0000131783.74034.97 15142859

[27] Jing S Gao X Yu B Qiao H. Evaluation of plaque characteristics in coronary artery patients with impaired glucose tolerance through optical coherence tomography. Rev Assoc Med Bras. (2018) 64:433–7. 10.1590/1806-9282.64.05.433 30304142

[28] Okon EB Chung AW Rauniyar P Padilla E Tejerina T McManus BM. Compromised arterial function in human type 2 diabetic patients. Diabetes. (2005) 54:2415–23. 10.2337/diabetes.54.8.2415 16046309

[29] Gao Y Lu B Sun ML Hou ZH Yu FF Cao HL. Comparison of atherosclerotic plaque by computed tomography angiography in patients with and without diabetes mellitus and with known or suspected coronary artery disease. Am J Cardiol. (2011) 108:809–13. 10.1016/j.amjcard.2011.04.032 21741605

[30] Gao X Song J Watase H Hippe DS Zhao X Canton G. Differences in carotid plaques between symptomatic patients with and without diabetes mellitus. Arterioscler Thromb Vasc Biol. (2019) 39:1234–9. 10.1161/ATVBAHA.118.312092 31070472

[31] Zwakenberg SR de Jong PA Hendriks EJ Westerink J Spiering W de Borst GJ. Intimal and medial calcification in relation to cardiovascular risk factors. PLoS ONE. (2020) 15:e0235228. 10.1371/journal.pone.0235228 32658909 PMC7357737

[32] Xu L Wang Z Liang Y Zhou C Fan T Yan J. Research on the significance of nuclear factor of activated t cells c1 with intravascular ultrasound in evaluating the risk of coronary heart disease. Chin J Arteriosc. (2016) 24:1115–8 (in Chinese).

[33] Yang H Yan J Su H Yuan W Xu L. The effect of intervention CD137-CD137 ligand axis on the c1 expression of nuclear factors in activated T of apolipoprotein E knockout mice. Chin J Cardiol. (2012) 40:775–9. 23141092

[34] Zhong W Yan J Wang Z. CD137 regulates the expression of apolipoprotein E~ (-/-) mouse nuclear factor of activated T C1 through microrNA-145A-5P. Chin J Cardiol. (2015) 43:887–93 (in Chinese). 26652991

[35] Yin Y Yan J Wang Z Liu P Liang Y. CD137 signal affects nuclear factor of activated T cell 1 expression of mouse aortic smooth muscle cells through nuclear factor kappa B. Chin J Cardiol. (2015) 43:614–8 (in Chinese). 26420323

[36] Liu Y Yan J Zhong W Xu L Liang X Wang Z. The function of micro RNA-124-2 on CD137-CD137L interaction regulating nuclear factor of activated T Cells C1 in mouse vascular smooth muscle cells. Chin J Arteriosc. (2016) 24:13–17 (in Chinese).

[37] Xu Y Wang Z Zhong W Shao C Li B Liu J. CD137-CD137L signal regulates the expression of nuclear factor of activated T cell c1 in mouse vascular smooth muscle cells via TRAF6/JNK/AP-1 pathway. Chin J Arteriosc. (2017) 25:7–12 (in Chinese).

[38] Weng J Yan J Chen Y Wang Z Wang C Shao C. CD137-CD137L signaling pathway promotes angiogenesis in mouse atherosclerotic plaques by c1 nuclear factor of activated T cell c1. Chin J Cardiol. (2016) 44:1040–6. 10.3760/cma.j.issn.0253-3758.2016.12.010 28056236

[39] Xu L Yan J Wang B Liu P Gong J Wang C. Expression and significance of NFATc1 and OX40-OX40L in atherosclerosis in ApoE~(-/-) mice. Jiangsu Med. (2011) 1384–7. 10.19460/j.cnki.0253-3685.2011.12.007 (in Chinese).

[40] Yan Y Zhang J Weng J Zang G Guo D Yang Y. The effect of OX40-OX40L interaction on the expression of NFATc1 and formation of the atherosclerotic plaques. Chin J Arteriosc. (2016) 24:1189–94 (in Chinese).

[41] Nilsson J Nilsson LM Chen YW Molkentin JD Erlinge D Gomez MF. High glucose activates nuclear factor of activated T cells in native vascular smooth muscle. Arterioscler Thromb Vasc Biol. (2006) 26:794–800. 10.1161/01.ATV.0000209513.00765.13 16469950

[42] Zhang Y Ma KL Gong YX Wang GH Hu ZB Liu L. Platelet microparticles mediate glomerular endothelial injury in early diabetic nephropathy. J Am Soc Nephrol. (2018) 29:2671–95. 10.1681/ASN.2018040368 30341150 PMC6218868

[43] Shan K Liu C Liu BH Chen X Dong R Liu X. Circular noncoding RNA HIPK3 mediates retinal vascular dysfunction in diabetes mellitus. Circulation. (2017) 136:1629–42. 10.1161/CIRCULATIONAHA.117.029004 28860123

[44] Meza CA La Favor JD Kim DH Hickner RC. Endothelial dysfunction: is there a hyperglycemia-induced imbalance of NOX and NOS? Int J Mol Sci. (2019) 20:3775. 10.3390/ijms20153775 31382355 PMC6696313

[45] Zetterqvist AV Berglund LM Blanco F Garcia-Vaz E Wigren M Dunér P. Inhibition of nuclear factor of activated T-cells (NFAT) suppresses accelerated atherosclerosis in diabetic mice. PLoS ONE. (2013) 8:e65020. 10.1371/journal.pone.0065020 23755169 PMC3670844

[46] Suehiro J Hamakubo T Kodama T Aird WC Minami T. Vascular endothelial growth factor activation of endothelial cells is mediated by early growth response-3. Blood. (2010) 115:2520–32. 10.1182/blood-2009-07-233478 19965691 PMC2845904

[47] Govatati S Pichavaram P Janjanam J Zhang B Singh NK Mani AM. NFATc1-E2F1-LMCD1-mediated IL-33 expression by thrombin is required for injury-induced neointima formation. Arterioscler Thromb Vasc Biol. (2019) 39:1212–6. 10.1161/ATVBAHA.119.312729 31043075 PMC6540998

[48] Wang Y Hu J Liu J Geng Z Tao Y Zheng F. The role of Ca^2+^/ NFAT in dysfunction and inflammation of human coronary endothelial cells induced by sera from patients with Kawasaki disease. Sci Rep. (2020) 10:4706. 10.1038/s41598-020-61667-y 32170198 PMC7069934

[49] Garcia-Vaz E McNeilly AD Berglund LM Ahmad A Gallagher JR Dutius Andersson AM. Inhibition of NFAT signaling restores microvascular endothelial function in diabetic mice. Diabetes. (2020) 69:424–35. 10.2337/db18-0870 31806622

[50] Ma XD Chen ZA Zheng JP. Phenotypic modulation of vascular smooth muscle cells in diabetes mellitus and intervention of traditional Chinese medicines. Zhongguo Zhong Yao Za Zhi. (2014) 39:4723–7 (in Chinese). 25898567

[51] Zeng M Luo Y Xu C Li R Chen N Deng X. Platelet-endothelial cell interactions modulate smooth muscle cell phenotype in an in vitro model of type 2diabetes mellitus. Am J Physiol Cell Physiol. (2019) 316:C186–97. 10.1152/ajpcell.00428.2018 30517030

[52] Mancarella S Potireddy S Wang Y Gao H Gandhirajan RK Autieri M. Targeted STIM deletion impairs calcium homeostasis, NFAT activation, and growth of smooth muscle. FASEB J. (2013) 27:893–906. 10.1096/fj.12-215293 23159931 PMC3574286

[53] Zhong W Li B Yang P Chen R Wang C Wang Z. CD137-CD137L interaction modulates neointima formation and the phenotype transformation of vascular smooth muscle cells via NFATc1signaling. Mol Cell Biochem. (2018) 439:65–74. 10.1007/s11010-017-3136-4 28770466

[54] Orr AW Lee MY Lemmon JA Yurdagul A Jr. Gomez MF Bortz PD. Molecular mechanisms of collagen isotype-specific modulation of smooth muscle cell phenotype. Arterioscler Thromb Vasc Biol. (2009) 29:225–31. 10.1161/ATVBAHA.108.178749 19023090 PMC2692987

[55] Cheng Y Liu X Yang J Lin Y Xu DZ Lu Q. MicroRNA-145, a novel smooth muscle cell phenotypic marker and modulator, controls vascular neointimal lesion formation. Circ Res. (2009) 105:158–66. 10.1161/CIRCRESAHA.109.197517 19542014 PMC2728297

[56] Yan J Yin Y Zhong W Wang C Wang Z. CD137 regulates NFATc1 expression in mouse VSMCs through TRAF6/NF-κB p65 signaling pathway. Mediators Inflamm. (2015) 2015:639780. 10.1155/2015/639780 26600673 PMC4639649

[57] Pang X Sun NL. Calcineurin-NFAT signaling is involved in phenylephrine-induced vascular smooth muscle cell proliferation. Acta Pharmacol Sin. (2009) 30:537–44. 10.1038/aps.2009.28 19349967 PMC4002815

[58] Shiny A Regin B Mohan V Balasubramanyam M. Coordinated augmentation of NFAT and NOD signaling mediates proliferative VSMC phenotype switch under hyperinsulinemia. Atherosclerosis. (2016) 246:257–66. 10.1016/j.atherosclerosis.2016.01.006 26814423

[59] Rendra E Riabov V Mossel DM Sevastyanova T Harmsen MC Kzhyshkowska J. Reactive oxygen species (ROS) in macrophage activation and function in diabetes. Immunobiology. (2019) 224:242–53. 10.1016/j.imbio.2018.11.010 30739804

[60] Frei B Higdon JV. Antioxidant activity of tea polyphenols in vivo: evidence from animal studies. J Nutr. (2013) 133:3275–84S. 10.1093/jn/133.10.3275S 14519826

[61] Krishnan TR Velusamy P Srinivasan A Ganesan T Mangaiah S Narasimhan K. EGCG mediated downregulation of NF-AT and macrophage infiltration in experimental hepatic steatosis. Exp Gerontol. (2014) 57:96–103. 10.1016/j.exger.2014.05.008 24844145

[62] Zavaczki E Gáll T Zarjou A Hendrik Z Potor L Tóth CZ. Ferryl hemoglobin inhibits osteoclastic differentiation of macrophages in hemorrhaged atherosclerotic plaques. Oxid Med Cell Longev. (2020) 2020:3721383. 10.1155/2020/3721383 32184915 PMC7063196

[63] Assar ME Angulo J Rodriguez-Manas L. Diabetes and ageing-induced vascular inflammation. J Physiol. (2016) 594:2125–46. 10.1113/JP270841 26435167 PMC4933100

[64] He S Zhang Y Huang S Yang L Li Y. The expression of interleukin 6 and its upstream signals in Xinjiang Kazakh patients with essential hypertension. J Clin Cardiol. (2016) 32:694–7. 10.13201/j.issn.1001-1439.2016.07.011 (in Chinese).

[65] Yan J Su H Xu L Wang C. OX40-OX40L interaction promotes proliferation and activation of lymphocytes via NFATc1in ApoE-deficient mice. PLoS ONE. (2013) 8:e60854. 10.1371/journal.pone.0060854 23593329 PMC3622016

[66] Mazière C Morlière P Massy Z Kamel S Louandre C Conte MA. Oxidized low-density lipoprotein elicits an intracellular calcium rise and increases the binding activity of the transcription factor NFAT. Free Radic Biol Med. (2005) 38:472–80. 10.1016/j.freeradbiomed.2004.10.028 15649649

[67] Nilsson-Berglund LM Zetterqvist AV Nilsson-Ohman J Sigvardsson M González Bosc LV Smith ML. Nuclear factor of activated T cells regulates osteopontin expression in arterial smooth muscle in response to diabetes-induced hyperglycemia. Arterioscler Thromb Vasc Biol. (2010) 30:218–24. 10.1161/ATVBAHA.109.199299 19965778 PMC2823568

[68] Wu Z Zhang Z Lei Z Lei P. CD14: biology and role in the pathogenesis of disease. Cytokine Growth Factor Rev. (2019) 48:24–31. 10.1016/j.cytogfr.2019.06.003 31296363

[69] van den Oord SC Akkus Z Renaud G Bosch JG van der Steen AF Sijbrands EJ. Assessment of carotid atherosclerosis, intraplaque neovascularization, and plaque ulceration using quantitative contrast-enhanced ultrasound in asymptomatic patients with diabetes mellitus. Eur Heart J Cardiovasc Imaging. (2014) 15:1213–8. 10.1093/ehjci/jeu127 24972806

[70] Xiong L Li P Zhao BW. Evaluation of carotid plaque neovascularization in patients with diabetes mellitus by contrast-enhanced ultrasonography. J Huazhong Univ Sci Technolog Med Sci. (2014) 34:29–32. 10.1007/s11596-014-1227-y 24496675

[71] Weng J Wang C Zhong W Li B Wang Z Shao C. Activation of CD137 signaling promotes angiogenesis in atherosclerosis via modulating endothelial Smad1/5-NFATc1 pathway. J Am Heart Assoc. (2017) 6:e004756. 10.1161/JAHA.116.004756 28288971 PMC5524009

[72] Goettsch C Rauner M Hamann C Sinningen K Hempel U Bornstein SR. Nuclear factor of activated T cells mediates oxidised LDL-induced calcification of vascular smooth muscle cells. Diabetologia. (2011) 54:2690–701. 10.1007/s00125-011-2219-0 21701818

[73] Alexopoulos A Bravou V Peroukides S Kaklamanis L Varakis J Alexopoulos D. Bone regulatory factors NFATc1 and Osterix in human calcific aortic valves. Int J Cardiol. (2010) 139:142–9. 10.1016/j.ijcard.2008.10.014 19019468

[74] Chen NX Moe SM. Pathophysiology of vascular calcification. Curr Osteoporos Rep. (2015) 13:372–80. 10.1007/s11914-015-0293-9 26409849

[75] Wang N Cui X Yang P Xu Y Li B Zhong W. CD137 signal mediates calcification of mouse vascular smooth muscle cells via exosomes transmitting nuclear factor of active T cell c1. Chin J Arteriosc. (2018) 26:1194–200, 1244. (in Chinese).

[76] Yu H van Berkel TJ Biessen EA. Therapeutic potential of VIVIT, a selective peptide inhibitor of nuclear factor of activated T cells, in cardiovascular disorders. Cardiovasc Drug Rev. (2007) 25:175–87. 10.1111/j.1527-3466.2007.00011.x 17614939

[77] Minami T Miura M Aird WC Kodama T. Thrombin-induced autoinhibitory factor, down syndrome critical region-1, attenuates NFAT-dependent vascular cell adhesion molecule-1expression and inflammation in the endothelium. J Biol Chem. (2006) 281:20503–20. 10.1074/jbc.M513112200 16627481

[78] Hamada N Miyata M Eto H Shirasawa T Akasaki Y Nagaki A. Tacrolimus-eluting stent inhibits neointimal hyperplasia via calcineurin/NFAT signaling in porcine coronary artery model. Atherosclerosis. (2010) 208:97–103. 10.1016/j.atherosclerosis.2009.07.040 19682688

